# Genome-guided development of a bacterial two-strain system for low-temperature soil biocementation

**DOI:** 10.1007/s00253-025-13448-8

**Published:** 2025-03-18

**Authors:** Karol Ciuchcinski, Grzegorz Czerwonka, Przemyslaw Decewicz, Zofia Godlewska, Katarzyna Misiolek, Katarzyna Zegadlo, Michal Styczynski, Lukasz Dziewit

**Affiliations:** 1 Department of Environmental Microbiology and Biotechnology, Institute of Microbiology, Faculty of Biology, University of Warsaw, Warsaw, Poland; 2 Division of Microbiology, Institute of Biology, Faculty of Exact and Natural Sciences, Jan Kochanowski University, Kielce, Poland; 3 Department of Hydraulic Engineering and Hydraulics, Faculty of Building Services, Hydro and Environmental Engineering, Warsaw University of Technology, Warsaw, Poland

**Keywords:** Antarctica, Biocementation, Microbial-induced calcite precipitation, Mobilome, *Sporosarcina*, Urease

## Abstract

**Abstract:**

Degradation and erosion of soil is a significant threat to global food security and overall agricultural productivity. This issue is exacerbated by climate change and intensive human activity, meaning that the development of sustainable solutions for those problems is critical. Microbially induced calcite precipitation (MICP) offers a promising approach to stabilise soil particles; however, its applicability at low temperatures remains limited. In our study, we introduce a novel two-strain system combining the type strain for biocementation experiments, *Sporosarcina pasteurii* DSM 33, and *Sporosarcina* sp. ANT_H38, a novel, psychrotolerant strain obtained from the Antarctic. The novel strain enabled enhanced biocementation performance when combined with the type strain. Biocementation experiments showed a 3.5-fold increase in soil cohesion, while maintaining a similar internal friction angle compared to the type strain alone (10.7 kPa vs 34.12 kPa; 0.55 kPa for untreated soil). The increased cohesion significantly reduces susceptibility to erosion, offering a practical and sustainable solution. Furthermore, to better understand the mechanisms driving this process, we conducted a comprehensive bioinformatic analysis of the ANT_H38 genome, revealing unique cold-adaptive genes, as well as urease genes, which are evolutionarily distant from other *Sporosarcina* ureases. Those results provide valuable insights into the strain’s functional adaptations, particularly under low-temperature conditions. Overall, our study addresses a critical issue, offering a robust, nature-based solution that enhances soil resilience through MICP. Performed laboratory work confirms the potential of the system for real-world applications, while the comprehensive bioinformatic analysis provides the much needed context and information regarding the possible mechanisms behind the process.

**Key points:**

*• Antarctic Sporosarcina sp. ANT_H38 contains unique urease genes*

*• Two-strain ANT_H38/DSM33 system effectively stabilises soil at low temperatures*

*• Two-strain system has potential for stopping soil erosion and desertification*

**Supplementary Information:**

The online version contains supplementary material available at 10.1007/s00253-025-13448-8.

## Introduction

Soil degradation and desertification pose significant threats to worldwide ecosystems, as well as agricultural productivity. The scale of the process is enormous, with over 1.5 billion hectares of land affected, constituting over 15% of the total land area on Earth. Therefore, addressing these issues is critical and requires sustainable solutions, including both restoration of soil properties, as well as preventing further deterioration. Among the most promising technologies is microbially induced calcite precipitation (MICP), a process involving the production of calcium carbonate by bacteria. When occurring in the environment, the CaCO_3_ precipitate can effectively bind loose particles, like sand grains, resulting in the formation of biocement. This phenomenon shows great potential for improving soil properties such as shear strength, compressive strength, and liquefaction resistance (Naveed et al. 2020; Lin et al. 2020). It has also been shown to increase the resilience of soils and minerals to naturally occurring weathering (Van Paassen et al. 2010; Matsubara 2021). Furthermore, the process of biocementation via MICP can serve as an effective carbon sequestration mechanism, incorporating atmospheric CO_2_ particles into the biocement (Fang and Achal 2024; Gilmour et al. 2024). Furthermore, MICP has found uses in processes such as bioremediation, wastewater treatment, production of self-healing concrete and more (Song et al. 2022; Zhang et al. 2023; Fouladi et al. 2023). Unfortunately, despite its many advantages, MICP faces many limitations as well (Ezzat 2023). Among those, temperature is one of the most prominent limiting factor, with majority of urease enzymes, critical for the process, being most active in temperatures between 20 and 28 °C (De Muynck et al. 2013; Anbu et al. 2016). This prevents MICP from being applied in cold and moderate environments, where the temperatures often drop below 10 °C.

To find a solution to this obstacle, we decided to explore one of the coldest biomes on Earth — the Antarctic. The extreme conditions found in Antarctica make it an ideal source of biological resources for bioprospecting, understood as exploration of environments in search of new molecules and enzymes. This is in line with the current interest in biotechnology of enzymes that exhibit new activities or can efficiently operate at low temperature. This interest stems not only from the need for enzymes that work in colder climates, but also from the fact that performing large-scale biotechnological processes at low temperatures is considered generally beneficial, as cost-effective approach frequently resulting in greater reaction efficiency by reducing the amount of undesired side reactions occurring (Siddiqui 2015). So far, cold-active enzymes have found their uses in many industries, with food processing and molecular biology being the biggest markets for new applications. Their thermal lability allows for convenient inactivation after a specific step in the process is performed. A prime example of leveraging this property can be seen in the dairy industry where β-D-galactosidases (EC 3.2.1.23) are commonly used to make lactose-free products. Similarly, alkaline phosphatases (EC 3.1.3.1) are often used in molecular biology laboratories to prevent undesired self-circularisation and concatenation of DNA (Ullrich et al. 1977; Karasová et al. 2002; Horner et al. 2011). In addition to food processing and molecular biology, cold-active enzymes have potential applications in other industries such as the production of detergents, pharmaceuticals and cosmetics (Angelaccio et al. 2012; Wang et al. 2018a, b).

*Sporosarcina* sp. ANT_H38 is an Antarctic bacterium of the genus *Sporosarcina*, belonging to the *Caryophanaceae* family, which was first described in 1936 by Kluyver and van Niel, and later amended by Yoon et al*.* in 2001 (Yoon et al. 2001). Bacteria belonging to this genus are mostly aerobic heterotrophs, with some species exhibiting facultative anaerobic metabolism. *Sporosarcina* spp. are either rod-shaped or coccoid, form spores and are motile. Representatives of this clade have been found in many different environments, such as soils, animal microbiomes, seawater and even human blood (Kwon et al. 2007; Tominaga et al. 2009; Gilroy et al. 2021; Bharti et al. 2023). The most prominent feature of many *Sporosarcina* strains is their ability to perform MICP, with *Sporosarcina pasteurii* being one of the model strains for this process (Lapierre et al. 2020). Considering this fact, as well as the Antarctic origin of the ANT_H38 strain, we believed this strain to be a strong candidate for enabling biocementation of soils at low temperatures. This could facilitate the application of this technique during colder seasons in temperate climates, extending its usability beyond the typical warmer seasons. Additionally, this opens the possibility for applications in more harsh environments, such as the Gobi Desert, or Greenland, both of which face ongoing desertification (Heindel et al. 2019).

In this study, we performed a complex genomic characterisation of novel bacterial isolate — cold-active, Antarctic *Sporosarcina* sp. ANT_H38. This enabled insight into its mobilome and metabolic potential. In its genome, a urease-encoding gene cluster was identified and the strain was tested for its ability to induce calcite precipitation as a factor increasing the shear strength, compressive strength, stiffness and liquefaction resistance of soil after the biomineralization process.

## Materials and methods

### Bacterial strains

*Sporosarcina* sp. ANT_H38 was previously isolated from the soil samples collected in 2012 from King George Island (Antarctica; GPS coordinates: 62 09.6010 S, 58 28.4640 W) (Romaniuk et al. 2018). *Sporosarcina pasteurii* DSM 33 originated from the Leibniz Institute DSMZ German Collection of Microorganisms and Cell Cultures GmbH (Šovljanski et al. 2022).

### DNA sequencing

DNA sequencing was performed applying an Illumina MiSeq instrument in paired-end mode using a v3 chemistry kit. The capillary sequencing of PCR products using an ABI3730xl DNA Analyzer (Applied Biosystems, Waltham, USA) applying the primer walking technique was used to confirm the circularity of plasmids. Additionally, DNA was also sequenced using the Oxford Nanopore technology to obtain long reads. A MinION MK1b device with R10.4.1 flowcell was used, along with matching chemistry sets. Raw data obtained from the device was basecalled using Guppy v.6.4.6 in High Accuracy mode.

### Genome assembly and annotation

The chromosome assembly process began with basecalled long reads. First, the data was filtered using Filtlong (version 0.2.1) with parameters: –keep_percent 90 and –min_len 1000 (github.com/rrwick/Filtlong). Following this, consensus assembly was conducted using Trycycler (version 0.5.3) (Wick et al. 2023). The assemblers employed in this step included Flye (version 2.9.1-b1784), Raven (version 1.8.1, github.com/lbcb-sci/raven) and Miniasm + minipolish (version 0.1.3) (Li 2016; Kolmogorov et al. 2019). Obtained contigs were polished using Medaka (version 1.7.2, modelr1041_e82_400bps_hac_h615) (github.com/nanoporetech/medaka), Polypolish (version 0.5.0) and POLCA (version 4.1.0) (Zimin and Salzberg 2020; Wick and Holt 2022). Illumina reads obtained from DNA sequencing were initially preprocessed with fastp (Chen et al. 2018).

Manual inspection of obtained contigs allowed the identification of four contigs representing plasmids. Obtained contigs were annotated using Bakta (v.1.6.1, https://github.com/oschwengers/bakta), database version 4.0. Annotations were then manually reviewed using the Artemis genome browser (v. 18.0.2, https://github.com/sanger-pathogens/Artemis).

### Functional annotation and core genome identification

Functional annotation of genes found in *Sporosarcina* sp. ANT_H38 was performed using the eggNOG-mapper v2 suite (version 2.1.12), utilising Prodigal for gene calling and DIAMOND for protein alignment (Hyatt et al. 2010; Huerta-Cepas et al. 2019; Buchfink et al. 2021; Cantalapiedra et al. 2021). The *emapper* command was run with default parameters, using the assembly as an input (–itype genome).

### Identification of cold-adapted genes

Proteins responsible for adaptation to cold environments were identified via a protein sequence-similarity-based approach, using blastp mode of DIAMOND sequence aligner version 2.0.15.153 (Buchfink et al. 2021). To obtain the best results, three separate reference databases were prepared. The first database was created by obtaining sequences of proteins responsible for cold adaptation described previously from the SwissProt database (Barria et al. 2013). This database is further referred to as the Cold Adaptation Database (CAD). The second database was also created as a subset of the SwissProt database, containing only sequences of cold shock proteins CspABCDEFHI, and is further referred to as CSPdb. Finally, the CAPP database of cold-adapted predicted proteins was used as the third reference database (Varliero et al. 2021). Only hits with at least 70% bi-directional coverage and identity were considered.

### Identification of mobile genetic elements

The following tools and databases were used to identify mobile genetic elements in genome assembly: PhiSpy v4.2.21, Prokaryotic virus Remote Homologous Groups (PHROGs) database and ViPTree for prophage regions (Akhter et al. 2012; Nishimura et al. 2017; Terzian et al. 2021); ISfinder for insertion sequences (Siguier et al. 2006); INTEGRALL database for integrons, integrases and gene cassettes (Moura et al. 2009); ICEFinder for integrative and conjugative elements (ICE) (Wang et al. 2024); MobileElementFinder for MITEs, insertion sequences, simple and composite transposons, ICEs, IMEs and CIMEs (Johansson et al. 2021). All gathered results were manually curated and validated using the Artemis genome browser (v. 18.0.2, NCBI Blast suite) and HHpred (Camacho et al. 2009; Carver et al. 2012; Zimmermann et al. 2018).

### Comparative genomic analysis

To determine the core genome between the ANT_H38 strain and other *Sporosarcina* species, the RIBAP pipeline (v1.0.3) was used (https://github.com/hoelzer-lab/ribap) (Lamkiewicz et al. 2024). First, assembled genomes were downloaded from the NCBI Genome webpage. Next, the RIBAP pipeline was ran with default parameters, utilising tools and databases such as Roary, Prokka, MMSeqs2, ILPs, MAFFT, FastTree, cd-hit, IQ-TREE and UpSetR (Price et al. 2010; Fu et al. 2012; Katoh and Standley 2013; Seemann 2014; Martinez et al. 2015; Page et al. 2015; Conway et al. 2017; Steinegger and Söding 2017; Minh et al. 2020). Gene sets with less than 20 genes were excluded from further analysis. For in-depth analysis of resulting gene sets, appropriate protein sequences were extracted from prokka output files and re-annotated using eggNOG-mapper v2 suite (v2.1.12), with default parameters and protein input (–itype proteins).

For building a graphical representation of similarity searches of plasmids and prophages, the clinker tool (version 0.0.27) was used (Van Den Belt et al. 2023).

### Evolutionary relationships analysis

Complete bacterial genomes and the *ureA* genes used to generate phylogenomic and phylogenetic trees were obtained from NCBI and extracted manually. Phylogenomic tree was generated using UBCG2 pipeline (Kim et al. 2021). For the *ureA*-based tree, obtained gene sequences were aligned using the Clustal Omega webserver (https://www.ebi.ac.uk/Tools/msa/clustalo/). Obtained multiple sequence alignment (MSA) was then used to build phylogenetic trees using raxml-ng (version 1.2.0), with the following parameters: –all –model TrN + I + G4 –bs-trees 2000 (Kozlov et al. 2019). The evolutionary model used was decided based on the results of analysis performed with modeltest-ng (version 3.22.1), according to the Bayesian information criterion for both alignments (Darriba et al. 2020). The R library *dendextend* was used to generate tanglegrams (Galili 2015). Finally, ANI and dDDH values were calculated using pyANI with default parameters (Meier-Kolthoff et al. 2022).

### Bacteria and cultivation conditions

*Sporosarcina pasteurii* DSM 33 and *Sporosarcina* sp. ANT_H38 were deposited in 8% DMSO stock solution of LB medium, supplemented with 1% (w/v) of filter sterilised urea (Sigma-Aldrich, Saint Louis, Missouri, USA) and frozen at − 70 °C for long-term storage. Most experiments were carried out at temperatures of 10 °C, 15 °C and 30 °C, with the exception of biomineralization, which was only performed at 10 °C.

### Growth kinetics

To determine and compare the ability of both strains to grow at low temperature, growth kinetics curves were determined at 10 °C and 15 °C. All cultures were grown in sterile glass tubes closed with cotton swabs, and LB supplemented with 1% urea was used as a growth medium. Growth rates were measured by determining the turbidity of each culture using the Biosan DEN-1B densitometer (Biosan, Riga, Latvia). Each tube was vortexed (15 s, 240 rpm) before the measurement to avoid bacterial precipitation. The measurements were carried out daily for 7 days, and each measurement was performed in triplicate.

### Utilisation of carbon sources

The ability of both *Sporosarcina* strains to utilise various carbon sources was tested using modified Spizizen’s minimal medium (SMM). Compared to the original medium, the 0.5% glucose (used as a sole carbon source) was replaced by glycerol, arabinose, lactose, maltose, trehalose, starch or sodium acetate (0.5% w/v each) (Kuhlmann and Bremer 2002). For each growth test, the medium was supplemented with L-cysteine, L-histidine, D-biotin, ornithine, proline, nicotinic acid, aspartic acid (20 mg/l each) and urea (1% w/v). Resazurin (0.02 mg/ml) was used as a growth indicator, with its colour changing as pH levels decreased below 6.5 (Borkowski et al. 2018). All media were filter sterilised (pore size 0.22 µm). Overnight cultures conducted in LB medium with 1% of urea were used as inoculants (1:100). Inoculated media with different carbon sources were incubated overnight at 30 °C without shaking. Samples where media colour changed from blue to pink were deemed positive for bacterial growth.

### Urease activity in biofilm

The ureolytic activity and biofilm formation capabilities of ANT_H38 and DSM 33 strains were tested on sand grains. First, sand grains were evenly layered in sterile cell culture inserts with medium-permeable membranes. These inserts were inserted into 6-well plates containing LB medium supplemented with 1% urea and inoculated with bacterial cultures, and then incubated at 10 °C for 7 days. The inserts were then removed and the medium containing bacteria was diluted in a 1:100 ratio. Following that, the inserts were reintroduced into the medium and incubated for 7 more days to promote biofilm formation. Finally, the inserts were transferred to a fresh medium containing 1 M urea, 0.5 M CaCl_2_ and 0.012 g/l phenol red indicator, with pH adjusted to 6.8. Absorbance measurements at 560 nm were taken at 60-min intervals using an Infinite 200 PRO Reader (Tecan, Männedorf, Switzerland) placed in a laboratory refrigerator (Pol-Eko, Wodzislaw Slaski, Poland) set to 10 °C.

### Biomineralization of sand samples and sand boxes biocementation tests

To conduct geotechnical tests on biomineralized sand, custom 3D-printed inserts, compatible with shear force measurement apparatus (80 × 80 × 50 mm external, 60 × 60 × 45 mm internal), were designed. The boxes were UV-sterilised for 30 min before use. Each box was filled with 200 g of autoclaved medium sand (121 °C, 15 min) and then inoculated with 50 ml of 7-day-old cultures of DSM 33 and/or ANT_H38 strains, previously cultured in LB medium with 1% (w/v) filter-sterilised urea, as well as *E. coli* DH5α (urease-deficient, negative control) grown in regular LB medium. Cultures were incubated at 10 °C for 14 days, with fresh urea-supplemented LB medium (10–20 ml) added every 48 h. For the experiment medium, sand was used, which was presented on sand grain size distribution curves (Supplementary File Figure S1). Sand was of marine origin, which determines the sphericity and roundness form of sand grains (Supplementary File Figure S2). The sand sample (200 g) was dried at 110 °C for 24 h before strain(s) injection.

Following the cultures’ incubation, a cementation medium (73.51 g/l calcium chloride dihydrate, 10 g/l urea, 20 g/l ammonium chloride, 2.12 g/l sodium bicarbonate, 6 g/l nutrient broth) was introduced every 48 h for over 2 weeks (Spencer et al. 2020). Each time, the medium was added until overfill (between 10 and 20 ml). Following the biocementation, the boxes were stabilised at room temperature for 20 days to reduce moisture content.

**Fig. 1:**
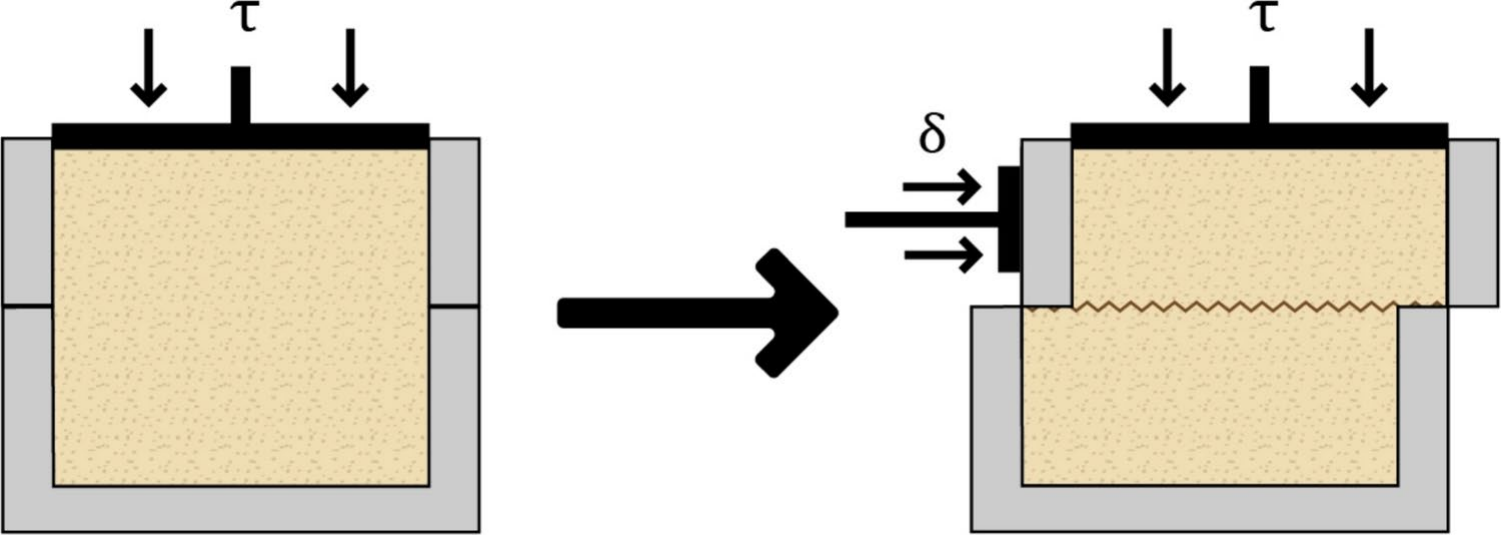
Principle of operation of the direct shear test. The apparatus consists of a rectangular box split horizontally into two halves. The test material (soil or sand) is placed within the box. A constant vertical load (τ) is applied to the top of the sample. A horizontal shear force (δ) is then applied to the upper half of the box, while the lower half remains fixed. The shear force at failure is recorded and used in conjunction with the applied vertical load to calculate the shear strength parameters: internal friction angle and cohesion

The primary objective of the performed experiment was to determine two key parameters: the internal friction angle and soil cohesion. The test involved applying a normal force to the upper part of a shear box within a direct shear apparatus, which directly reflects Coulomb’s law. The shear box consisted of two parts: the lower part was fixed within the apparatus, while a horizontal shear force (τ) was applied to the upper part, which was simultaneously subjected to a vertical normal force (δ). The application of shear force to the side of the box caused the failure of interparticle bonds within the tested material. By measuring the shear force exerted on the side of the box and the normal force applied to its upper part, the internal friction angle and soil cohesion were determined (Fig. 1).

Internal friction angles and soil cohesion were measured following Polish standard PN-B-04481:1988. This laboratory method complies with ISO 17892, *Geotechnical Investigation and Testing — Laboratory Testing of Soil*, in accordance with European Standards EN 1997–1 and EN 1997–2. Normal stress values for strength tests were *σ* = 12.5, 25, 50 and 100 kPa, with three to five samples tested per bacterial strain or a pair of strains.

### Biosafety analysis

Potential antibiotic resistance genes were identified using NCBI Antimicrobial Resistance Gene Finder version 3.11.2 with database version 2024–01–31.1 (AMRFinderPlus; https://github.com/ncbi/amr), and via sequence similarity-based search against the CARD database v 3.2.5 (Alcock et al. 2023). Potential genes conferring metal resistance were identified through a sequence-similarity-based search versus the METGeneDb database (accessed 1.02.2023) (Dziurzynski et al. 2022). Additionally, results from the “STRESS” category indicating metal resistance from AMRFinderPlus search were also included. Similarly, the presence of virulence factors was determined by comparing protein sequences to the Virulence Factor Database (VFDB; accessed 26.04.2024) (Liu et al. 2021). All protein-vs-protein searches were performed using DIAMOND in blastp mode, and cutoff values for results were at least 70% identity and bi-directional coverage.

## Results

### Genome of *Sporosarcinasp*. ANT_H38 — general information

*Sporosarcina* sp. ANT_H38 was isolated from the petroleum-contaminated soil at the Henryk Arctowski Polish Antarctic Station in 2012 (GPS coordinates: 62°09.601′ S, 58°28.464′ W). The strain was able to grow in a wide range of temperatures, i.e., 4–37 °C, and in a pH ranging between 7 and 11 (Romaniuk et al. 2018).

The genomic DNA isolated from *Sporosarcina* sp. ANT_H38 was sequenced using both Oxford Nanopore and Illumina technologies and subsequently assembled into a single, circular chromosome and four plasmids (pA38H1-pA38H4). The genome has a total size of 4,685,507 bp with a GC content of 39.9%. Following the assembly, the genome was automatically annotated using Bakta version 1.6.1, and the general features of the ANT_H38 genome can be found in Supplementary File Table S1.

### Genome of *Sporosarcinasp*. ANT_H38 — functional annotation

**Fig. 2:**
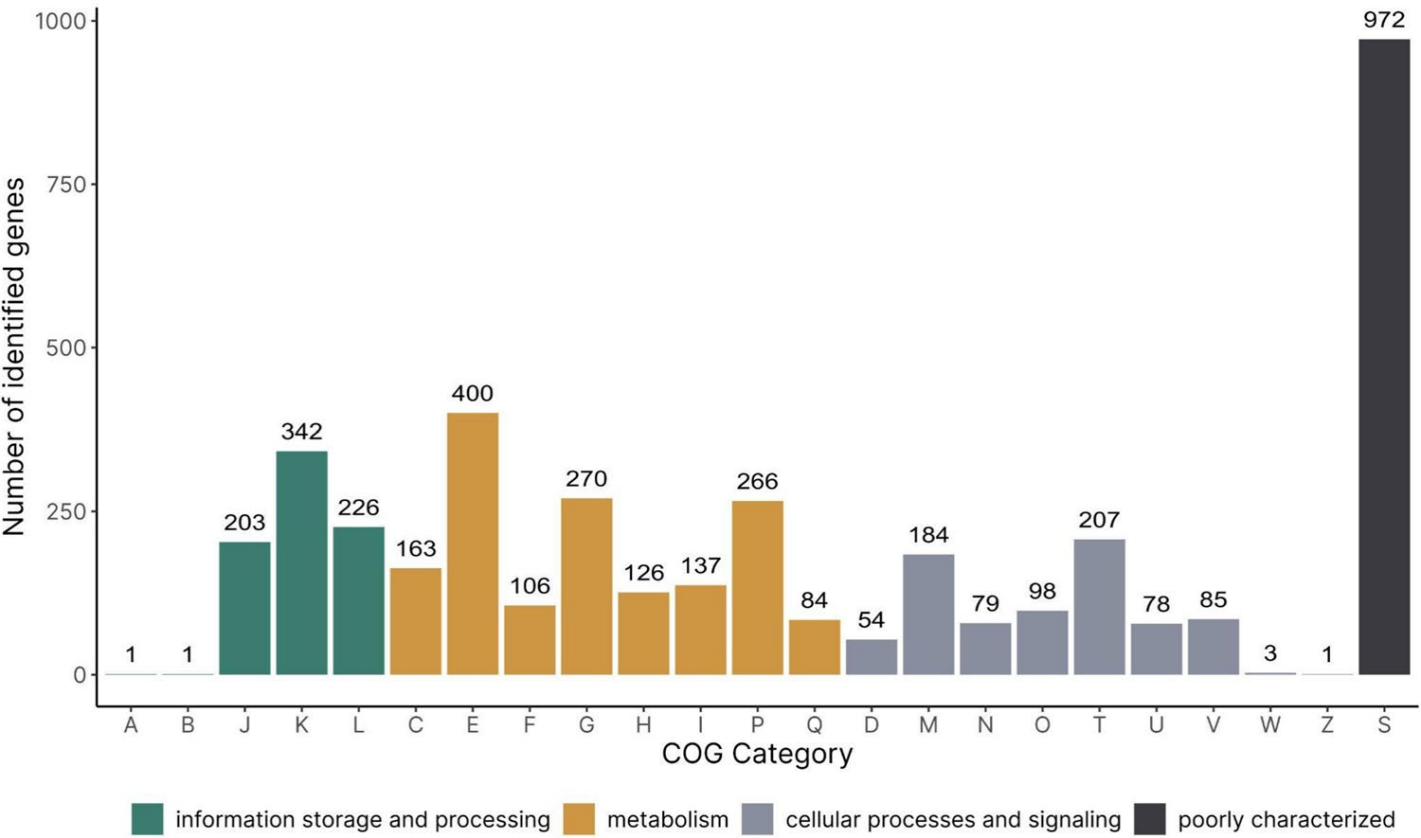
COG categories assigned to genes found within *Sporosarcina* sp. ANT_H38 and their respective counts. Annotations for COG categories are as follows: A: RNA processing and modification, B: Chromatin structure and dynamics, C: Energy production and conversion, D: Cell cycle control and mitosis, E: Amino Acid metabolism and transport, F: Nucleotide metabolism and transport, G: Carbohydrate metabolism and transport, H: Coenzyme metabolism, I: Lipid metabolism, J: Translation, K: Transcription, L: Replication and repair, M: Cell wall/membrane/envelope biogenesis, N: Cell motility, O: Post-translational modification, protein turnover, chaperone functions, P: Inorganic ion transport and metabolism, Q: Secondary Structure, T: Signal transduction, U: Intracellular trafficking and secretion, Y: Nuclear structure, Z: Cytoskeleton, R: General functional prediction only, S: Function unknown

Functional annotation of *Sporosarcina* sp. ANT_H38 was performed using eggNOG_mapper software. Genes related to cellular metabolism were the most abundant (1552 genes), followed by poorly characterised genes (972), genes involved in cellular processes and signalling (789) and information storage and processing (773). In terms of specific COG categories, amino acid transport and metabolism (E, 9.7%) and transcription (K, 8.3%) dominated, followed by transport and metabolism of carbohydrates (G, 6.6%), and inorganic ions (P, 6.5%) (Fig. 2).

### Cold adaptation features identified in the ANT_H38 genome

The genome of *Sporosarcina* sp. ANT_H38 has been extensively characterised to identify the genomic features responsible for its cold-adaptation capabilities. Protein sequence similarity analysis was performed using three separate reference databases, including sequences of proteins responsible for cold adaptation, cold shock proteins, and a database of cold-adapted predicted proteins CAPP (Barria et al. 2013). In total, 45 cold-adaptive genes were identified in the ANT_H38 genome. Of these, eight proteins matched to the Cold Adaptation Database CAD, including proteins involved in global DNA transcription regulation (DnaA, NusA), translation initiation (InfABC), and cold shock proteins (CspB, Pnp). Additionally, 35 proteins were hit to the CAPP database, including multiple 30S and 50S ribosomal proteins, and proteins involved in carbohydrate metabolism (i.e. succinate-CoA ligase [ADP-forming] subunit alpha, SucD; 1,4-dihydroxy-2-naphthoyl-CoA synthase, MenB). Furthermore, a search versus the CSPdb database of cold shock proteins revealed two additional copies of the cold shock protein CspA. All of these proteins play a role in the strain’s ability to survive in temperatures below freezing by regulating its metabolism (Ivancic et al. 2013). A full list of identified proteins was presented in Supplementary File Table S2.

### Mobilome of *Sporosarcinasp*. ANT_H38

Investigation of mobile genetic elements (MGEs) such as plasmids, transposable elements or (pro)phages provides insights into the mechanisms behind horizontal gene transfer, genomic evolution, and the acquisition of accessory genes (Tokuda and Shintani 2024). In this study, we delved into the *Sporosarcina* sp. ANT_H38 mobilome, expanding our knowledge of the strain’s genetic complexity.

**Fig. 3:**
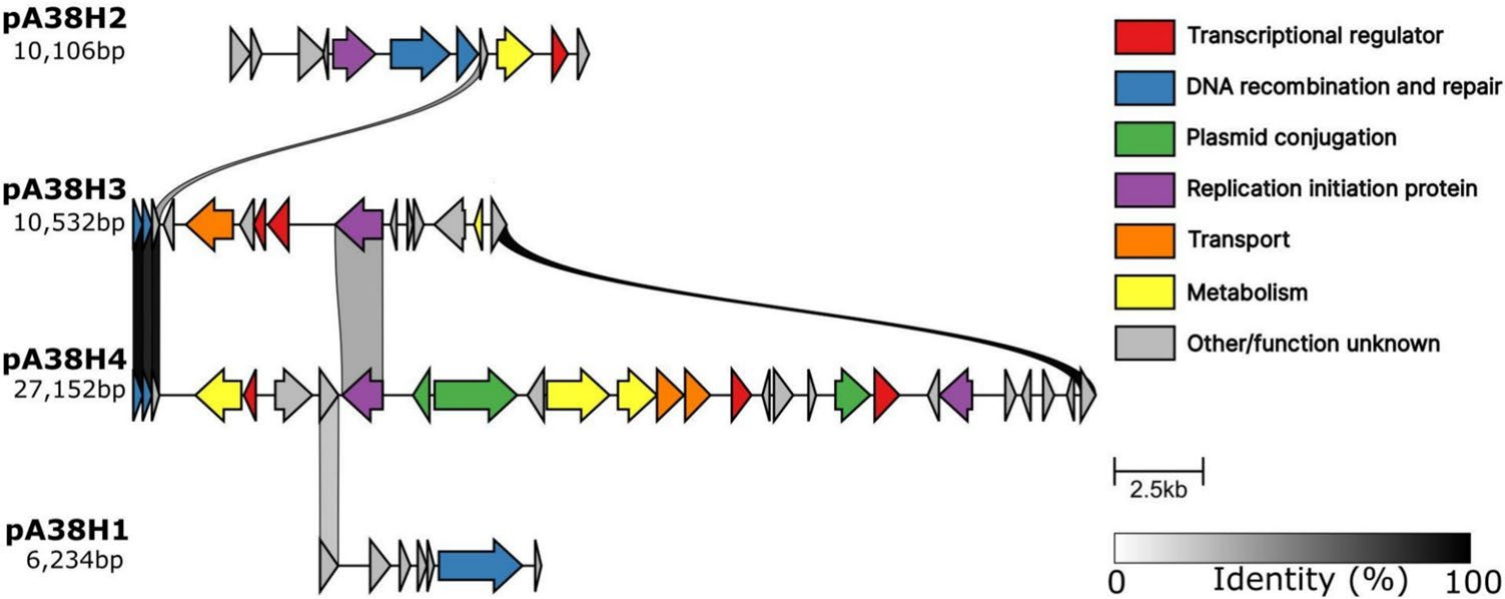
Linear presentation of the ANT_H38 plasmids. Each line represents a plasmid and each arrow corresponds to a protein-coding sequence. Arrows linked by a grey-scale block indicate at least 30% protein sequence similarity (determined by the clinker tool version 0.0.27). Protein colours correspond to their functional modules, as shown in the legend

In total, four plasmids with sizes of 6234 bp (pA38H1), 10,106 bp (pA38H2), 10,532 bp (pA38H3), and 27,152 bp (pA38H4) (Fig. 3) were identified. The GC content of these plasmids ranges from 33.3% (pA38H1) to 36.7% (pA38H3), which is significantly lower than the GC content (39.9%) of the chromosome. Outside of genes responsible for plasmid functioning and maintenance, i.e. replication (REP) and mobilisation for conjugal transfer (MOB), most sequences were only assigned a putative function. These genes were described in Supplementary File Table S3. Additionally, a visual representation of the plasmids, with key features marked, can be found in Fig. 3.

Comparison with plasmids deposited within the PLSDB database (version 2023_11_03_v2) revealed no significant similarity to any known plasmid sequence, pointing towards a distinct evolutionary origin.

Furthermore, a total of 15 transposase genes were identified within the *Sporosarcina* sp. ANT_H38 genome, representing families: IS*150* (2 elements), IS*1182* (2), IS*21* (1), IS*30* (1), IS*L3* (2). A summary of the identified transposases and their genomic locations is provided in the Supplementary File Table S4.

**Fig. 4:**
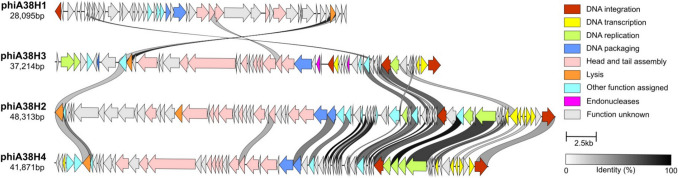
Linear representation of the ANT_H38 prophages. Each line represents a prophage and each arrow corresponds to a protein-coding sequence. Arrows linked by a grey-scale block indicate at least 30% protein sequence similarity (determined by the clinker tool version v0.0.27). Protein colours correspond to their functional modules, as shown in the legend

Finally, the analysis has revealed the presence of four tailed prophages within the genome, namely phiA38H1 (coordinates: 2,402,250 to 2,430,344; 28 kb in total; 38.26% G-C content), phiA38H2 (3,777,853 to 3,826,165; 48 kb; 41.61%), phiA38H3 (3,843,298 to 3,880,511; 37 kb; 40.12%) and phiA38H4 (4,345,518 to 4,387,388; 42 kb; 41.56%). We were able to identify perfect direct terminal repeats bordering phiA38H3 – AATCGGCACGAAATCGGCACGAA (23 bp), and phiA38H4 – ATTACATCATGCCGCCCAT (19 bp), both located in intergenic regions, which might act as attachment sites recognised by phages during their integration into host’s genome. This observation indicates that both prophages might be complete and functional. The comparison of their genomes revealed limited internal protein sequence-based similarity (Fig. 4).

Exploring the gene context of these prophages we observed that phiA38H3 has integrated near or within the iron-sulphur gene cluster (SufBCD), which may potentially impact host metabolism. Upstream of phiA38H4 lies a cluster of recombination-related proteins, including FtsK and a type I restriction-modification system. These may function in defending against foreign DNA and altering the host genome during prophage induction. Interestingly, this 29-kb region is flanked by a truncated attachment site (TTACATCATGCCGCCCA) missing one nucleotide on the 5′ end. Another tyrosine integrase (GGGNBK_21310) with low sequence identity (34%) to phiA38H4’s integrase (GGGNBK_21670) is also present, suggesting this region could be transduced independently or as part of the prophage genome.

### Comparative genomics of *Sporosarcinaspp*.

**Fig. 5:**
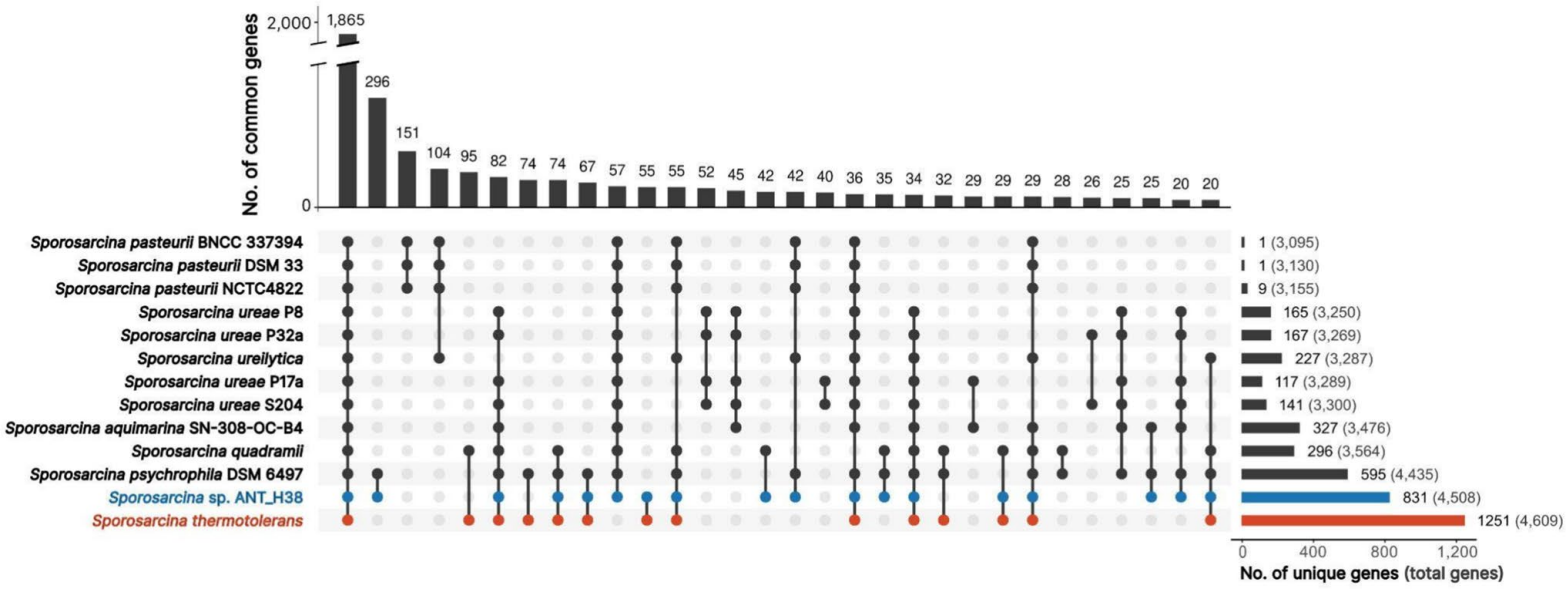
UpSet plot displaying the core genome and accessory genes for analysed *Sporosarcina* strains. Sets with less than 20 common genes were not displayed. The ANT_H38 strain was marked in blue, while *Sporosarcina thermotolerans*, as the strain with the most unique genes, was marked in red. The number of unique genes for each strain is shown on the bar plot on the right side of the figure, along with the total gene count in each genome (in parentheses)

To gain insight into the genomic diversity within the *Sporosarcina* genus a comparative genomic analysis was performed. Based on completeness of the genome, we selected 12 *Sporosarcina* strains from the NCBI genome database: *S. ureilytica* (GCF_001753205.1); *S. quadrami* (GCF_014836615.1); *S. thermotolerans* (GCA_033253685.1); *S. pasteurii* NCTC4822 (GCF_900457495.1), BNCC337394 (GCF_004379295.1), and DSM 33 (GCF_031822395.1); *S. psychrophila* DSM 6497 (GCF_001590685.1); *Sporosarcina* sp. ANT_H38 (GCF_008369195.1); *S. ureae* P32a (GCF_002109325.1), P17a (GCF_002082015.1), P8 (GCF_002101375.1), and S204 (GCF_002081995.1); *S. aquimarina* SN-308-OC-B4 (GCF_019748715.1). The RIBAP pipeline was used to determine core and accessory gene sets. Following this, genes either unique to specific strains or shared amongst multiple strains (Fig. 5) were extracted and re-annotated using eggNOG-mapper v2.

The core genome was estimated to be 1865 genes, comprising between 40.4% (*S. thermotolerans*) and 60.2% (*S. pasteurii* BNCC337394) of the entire genome. Strains with the highest number of unique genes were the ones occupying more specialised environmental niches, specifically *S. thermotolerans* (1251), *Sporosarcina* sp. ANT_H38 (831), and *S. psychrophila* DSM6497 (595). Furthermore, despite the low number of unique genes within each *S. pasteurii* strain (1, 1, and 9 for BNCC33794, DSM33, and NCTC4822 strains, respectively), all three share 151 genes not found in other genomes. In contrast, *S. ureae* strains exhibited a greater degree of intra-species diversity, with a higher number of unique genes (117–167), and only 52 unique within the species and shared between four strains. The biggest unique intersection (understood as genes shared by at least two strains that are unique to them) was observed for both psychrotolerant strains, *Sporosarcina* sp. ANT_H38 and *S. psychrotolerans.* This set includes genes responsible for glycogen synthesis and breakdown (*glgABDP*); foldase protein *psrA1*; multiple stress response proteins (*csbD*; *yceC* and two copies of *yceD*); carbohydrate transporters/permeases (*araQ*, *gntP, ugpA*); and lichenan-specific phosphotransferase system (*licABC*). Overall, the function of the aforementioned genes indicates adaptation for cold environments. This result further confirms the results of UBCG-based phylogenetic analysis, indicating the similarity between these two strains, and their distance from other analysed strains.

Furthermore, the set of genes unique to *Sporosarcina* sp. ANT_H38 was analysed. The most prominent feature of this set was the abundance of genes responsible for carbohydrate metabolism. These genes are summarised in Supplementary File Table S5. Other than that, we additionally identified six copies of *btuD* gene and a single copy of *btuF*, responsible for vitamin B12 import and binding; as well as the *uvrA* gene, involved in protection from UV radiation.

### Identification and phylogeny of the urease-encoding gene cluster

The production of urease in bacteria is well described, relying on the *ureABCDEFG* gene cluster. The first three genes, *ureA*, *ureB* and *ureC*, encode the three subunits of the urease enzyme, while the rest of genes have accessory functions, and are responsible for maturation and activation of the enzyme. To investigate the biotechnological potential of the ANT_H38 urease, we sought to understand its evolutionary relationship to ureases in other *Sporosarcina* strains. Here, we focus on the *ureA* gene, which encodes the large catalytic subunit.

A phylogenetic analysis was conducted to trace the evolution of the *ureA* gene across 13 *Sporosarcina* strains (the same strains were previously used for comparative genomic analysis), and *Planococcus* sp. PAMC_21323 (GCF_000785555.1) as an outgroup. First, we constructed a reference tree based on a comprehensive set of core bacterial genes using the UBCG2 pipeline (Kim et al. 2021). This tree largely confirmed the expected evolutionary relationships, with strains of the same species clustering together. Notably, the ANT_H38 strain clustered closely with *S. psychrophila*, reflecting their possible common evolutionary origin.

**Fig. 6:**
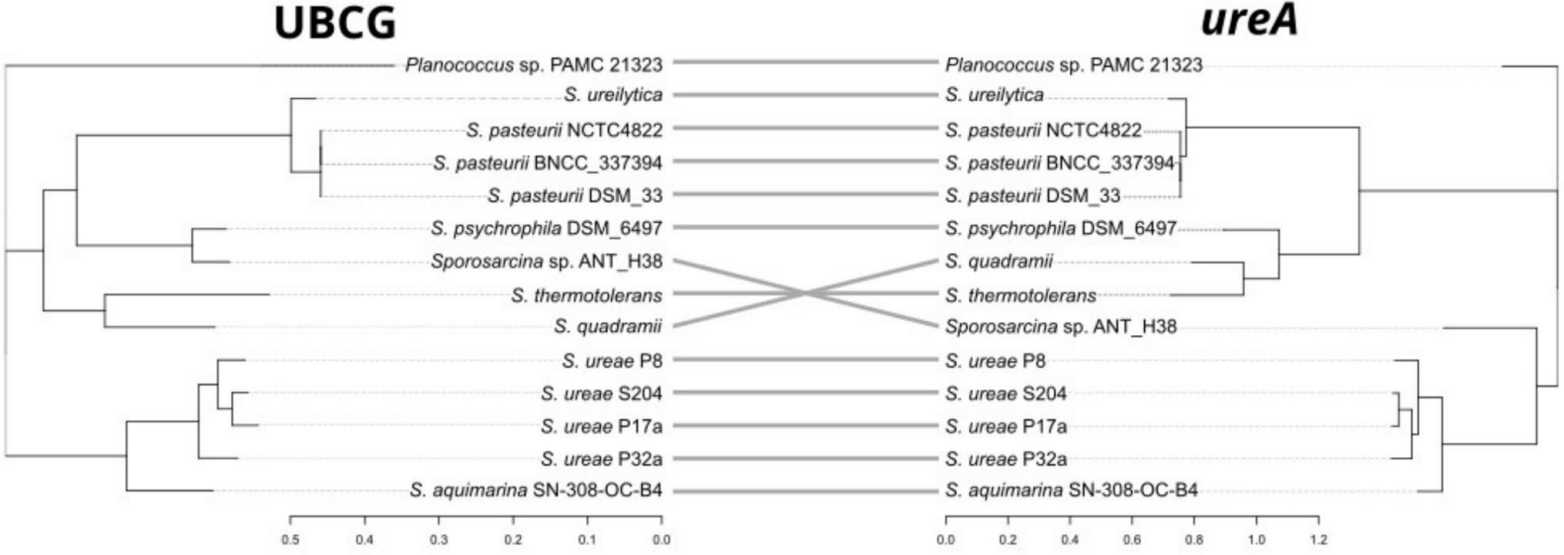
Comparison of evolutionary relationships between *Sporosarcina* strains as determined by UBCG-based (left) and *ureA*-based (right) analyses

Next, a similar analysis was performed using the *ureA* gene sequences. The *ureA*-based tree largely mirrored the UBCG tree, showing two major groups corresponding to *S. ureae/S. aquimarina* and *S. pasteurii/S. ureilytica* clusters. Interestingly, the ANT_H38 showed a notable deviation. While the phylogenomic analysis shows a close relationship between the ANT_H38 strain and *S. psychrophila*, the *ureA* phylogeny places the ANT_H38 near the root of the tree (Fig. 6). This suggests that the urease from this strain may be evolutionarily distinct from ureases in other *Sporosarcina* strains, including its close relative *S. psychrophila*.

Finally, to provide a greater insight into the relationship between the ANT_H38 strain, and its closest relative, *S. psychrophila*, we calculated average nucleotide identity (ANI) between their genomes, and performed digital DNA:DNA hybridisation (dDDH). Obtained results confirmed previous findings, with the ANI value reaching 88.26% (typically observed for closely related organisms of different species), and the dDDH was only 33.6%. This, combined with the results of comparative genomic analysis, presents solid evidence that despite sharing a relatively recent common ancestor, the two strains have undergone significant divergent evolution.

### Growth kinetics, biofilm formation and urease activity

**Fig. 7:**
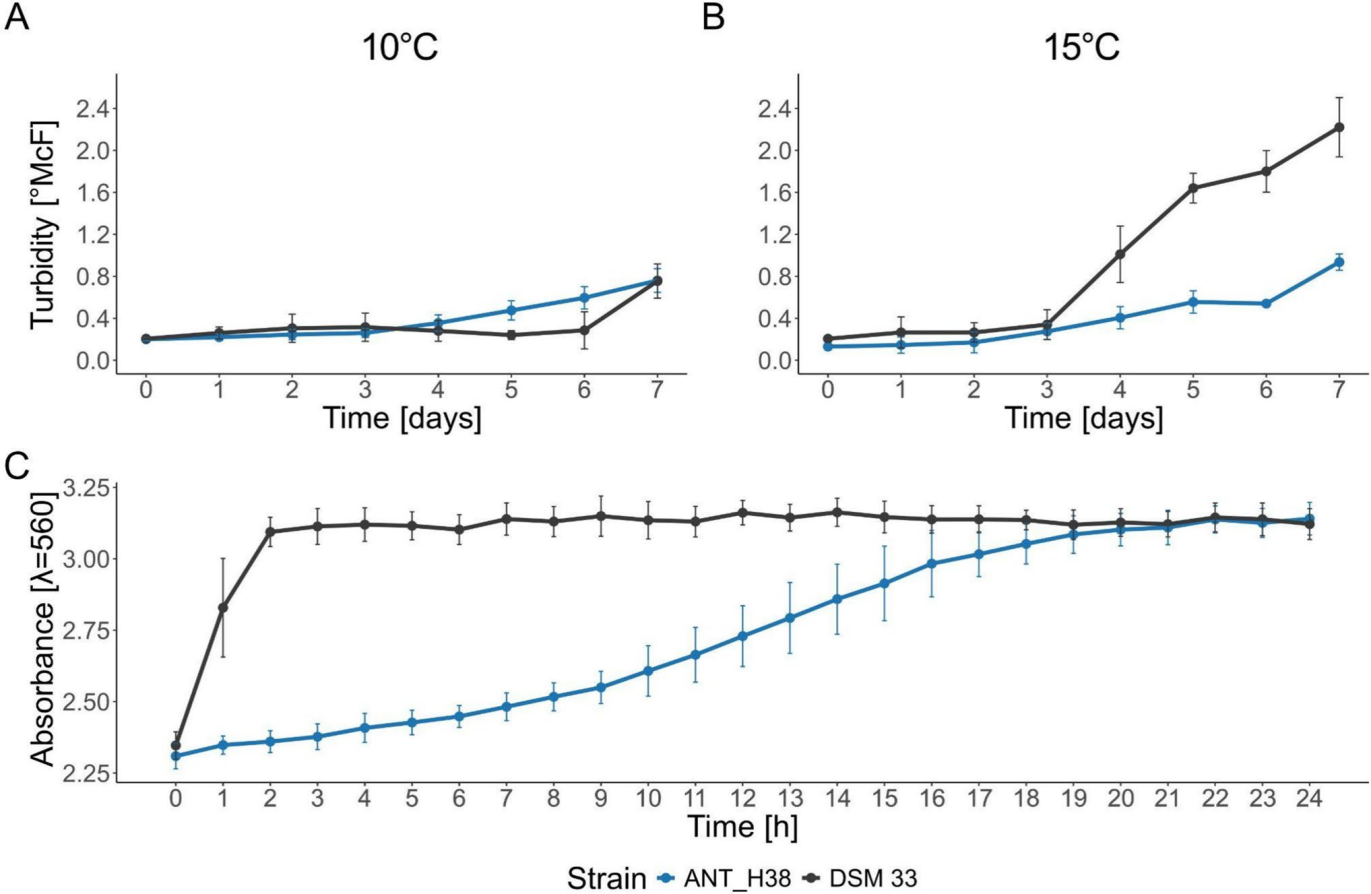
Comparison of growth kinetics of S. *pasteurii* DSM 33 and *Sporosarcina* sp. ANT_H38 at **A** 10 °C and **B** 15 °C over 7 days. **C** Urea hydrolysis kinetics by bacterial cells in biofilm formed on sand grains, measured by phenol red indicator absorbance change at 10 °C over 24 h

Both tested strains demonstrated growth at 10 °C and 15 °C. At 10 °C, both strains grew similarly until day 5 and 6, where *Sporosarcina* sp. ANT_H38 samples started exhibiting higher turbidity compared to *S. pasteurii* DSM 33. On day 7, measurements for both strains showed similar values (Fig. 7A). At 15 °C, the growth of both strains was again similar in the initial phase (first 3 days); however, starting from day 4, turbidity of DSM 33 samples was much higher compared to the ANT_H38 strain (Fig. 7B). The measurements indicated that both strains are able to multiplicate and persist in low-temperature environments, with *Sporosarcina* sp. ANT_H38 being slightly favoured at lower temperatures (10 °C), and *S. pasteurii* DSM 33 growing faster at higher temperature (15 °C).

Subsequently, biofilm formation and ureolytic activity were assessed via sand grain colonisation and subsequent urea hydrolysis, measured by pH changes via phenol red indicator. Both strains demonstrated biofilm formation capabilities and ureolytic activity. DSM 33 exhibited significantly higher ureolytic activity at 10 °C, confirming previous findings (Lapierre et al. 2020). ANT_H38 showed lower and delayed ureolytic activity compared to the type strain (Fig. 7C).

### Utilisation of various carbon sources

In the next step, the ability of both strains to metabolise various carbon sources was tested. The analysis revealed that the type strain *S. pasteurii* DSM 33 was able to metabolise sodium acetate, arabinose, trehalose and starch as sole carbon sources. *Sporosarcina* sp. ANT_H38 demonstrated a broader carbon utilisation profile, metabolising glycerol, lactose and maltose, in addition to carbon sources utilised by DSM 33. Conversely, the ANT_H38 strain was unable to metabolise sodium acetate. Those results fall in line with results of bioinformatic analyses, since genes putatively enabling the utilisation of these carbon sources were distinguished within the ANT_H38 genome.

### Biocementation analysis

The results of biocementation analysis demonstrated a significant increase in both internal friction angle and cohesion of sand samples treated with DSM 33 and 1:1 mixture of DSM 33 and ANT_H38, compared to untreated sand samples and those treated with urease-deficient *E. coli* DH5α (negative control). The addition of the ANT_H38 strain alone did raise the internal friction angle substantially, although not as much as other tested variants.

When assessing soil biocementation, both the internal friction angle (determining shear strength, a key characteristic of non-cohesive soils) and soil cohesion (reflecting intermolecular forces due to formed calcite bridges) are crucial parameters. It is important to note that the best combination of both of those factors was obtained when using a 1:1 mixture of tested strains. Notably, soil cohesion in this experimental setup was over three times higher compared to the reference strain alone, and almost five times higher than in the negative control (Table 1). Interestingly, when using the ANT_H38 strain alone, soil cohesion was almost as low as for bacteria-free sand beds. Overall, the results indicate a strong synergistic effect occurring between *S. pasteurii* DSM 33 and *Sporosarcina* sp. ANT_H38.

**Table 1:** Internal friction angle and soil cohesion parameters of biocement resulted from calcite precipitation by *S. pasteurii* DSM 33, ANT_H38, and both strains used in co-culture, compared to urease-depleted *E. coli* DH5α

Inoculation	Internal friction angle [*θ*]	Soil cohesion [kPa]	Coefficient of determination (*R*^2^)
*Sporosarcina* sp. ANT_H38	30.61	0.81	0.9533
*S. pasteurii *DSM 33	80.94	10.70	0.9737
*Sporosarcina* sp*. *ANT_H38 + *S. pasteurii *DSM 33	77.00	34.12	0.9701
*E. coli *DH5α	18.90	7.13	1.2000
Bacteria-free sand bed	38.48	0.55	0.9910

### Biosafety analysis

To ensure that the strain is safe to use in any potential biotechnological applications, a comprehensive analysis of the genome was performed. The analysis did not reveal the presence of any known antibiotic-resistance genes. While two copies of the *arsC* gene were identified, these are associated with toxic metal resistance and are neither directly relevant to any biosafety concerns. Further investigation using the VFDB yielded seven putative virulence-related proteins. Close inspection of these hits revealed that none of these proteins are considered primary virulence factors, i.e. are not sufficient on their own to provide virulence.

## Discussion

In this work, we characterised the *Sporosarcina* sp. ANT_H38 strain and explored its role as a potential enhancer strain in a two-component microbial-induced calcite precipitation system. The first step in this process was the reconstruction of a complete genomic sequence. To achieve this, we used multiple techniques, including hybrid Oxford Nanopore-Illumina sequencing, as well as primer-walking for circularity analysis of plasmid sequences. As a result, we were able to obtain a complete, circular chromosome sequence and sequences of four small plasmids. This allowed us to determine the metabolic potential of the strain, as well as its adaptive features, such as genes responsible for cold tolerance. Analysis of the whole genome also allowed us to determine that the strain is safe to use, as it contains no genes responsible for antibiotic resistance or virulence. We also thoroughly analysed the mobilome of this strain and performed one of the first complete annotations of mobile genetic elements within the *Sporosarcina* genus.

Amongst analysed MGEs, plasmids can offer significant evolutionary advantage to the host; thus, we were interested in what function is encoded within four plasmids of the ANT_H38 strain. We were able to identify *merR*-like transcriptional regulators on plasmids pA38H2, pA38H3 and pA38H4 (2 copies), hinting towards the role of metals and other stressors in regulation of expression of genes encoded within these elements (Brown et al. 2003). Additionally, genes involved in biofilm formation and sporulation were also found, expanding on the potential role of plasmids in adaptation to harsh environments. Notably, two genes encoding proteins putatively responsible for pH regulation (carbonate dehydratase and Ktr system potassium transporter, GGGNBK_23210 and GGGNBK_23180, respectively) are present in plasmid pA38H3. Their presence may be especially beneficial in the context of calcite precipitation, since the process releases ammonia, thus heavily increasing the pH of the environment (Seifan and Berenjian 2019).

The next step in the genome-guided analysis of biotechnological potential of the strain was to analyse the urease gene cluster, responsible for urea degradation and precipitation of calcite. To gain insights into the origin of this enzyme, as well as its similarity to other urease enzymes found in *Sporosarcina* strains, we performed a phylogenomic and phylogenetic analysis. Our first step was to create a reference tree based on core bacterial genes (Fig. 6). The tree was largely convergent with our expectations, mimicking the expected strain/species relationships. Next, we used the *ureA* gene, encoding the large catalytic subunit of the urease enzyme, to explore its similarity and relationship to other *Sporosarcina*-encoded ureases. Finally, we compared the results of both performed analyses. Overall, the obtained trees are almost identical, bearing the location of *Sporosarcina* sp. ANT_H38. This solidifies the use of *ureA* gene as a phylogenetic marker in urease-producing *Sporosarcina* strains. However, the key takeaway from the phylogenetic analysis is related to the position of *Sporosarcina* sp. ANT_H38 on the *ureA*-based tree. First of all, the strain is located on the outer branch of a bigger clade containing the *S. ureae* and *S. aquimarina* strains, separate from the *S. pasteurii*. This is important, given that *S. pasteurii* is the type strain for most biocementation and MICP-based experiments. Overall, the urease of the ANT_H38 strain seems to be quite different from other *Sporosarcina* strains, providing a promising platform for the exploration of its use in biotechnology.

Finally, we thoroughly investigated the potential of *Sporosarcina* sp. ANT_H38 for biocementation. Previously, the reference strain used in this study — *S. pasteurii* DSM 33 — alongside other *Sporosarcina* and *Bacillus* strains, were successfully used as biocementing factors (Dapurkar and Telang 2017). However, most previously used strains are unable to grow at low temperatures, making it difficult to apply the technologies during winter seasons in a temperate climatic zone. To overcome this challenge, we investigated *Sporosarcina* sp. ANT_H38 as a psychrotolerant, urease-producing strain that has the potential to biocement soils even at low temperatures.

First, we looked into the growth of both ANT_H38 and DSM 33 strains at low temperatures. Our results indicate that while at 15 °C the type strain grows faster, the ANT_H38 strain takes over as the temperature drops. At 10 °C, the psychrotolerant strain takes only 3 days to start its rapid growth, while the type strain requires 6 days to do so. This difference may be significant in the scope of biotechnological applications, potentially leading to reduction of overall time needed to achieve the same result.

Next, we tested the urease activity of both strains. In general, the urease of *Sporosarcina pasteurii* DSM 33 seemed to perform better compared to the ANT_H38 strain, reaching its peak activity after just 2 h. Urease of ANT_H38 did eventually reach the same level of activity, but it required 20 h.

Finally, we looked into the most important parameter in the scope of biotechnological application of the strain — biocementation performance. At this point, it became clear that the ANT_H38 strain is unable to effectively biocement sandy soils on its own. Compared to the type strain, it performed much worse both in terms of increasing the internal friction angle (30.6° vs 80.9°) and soil cohesion (0.81 vs 10.70). This could be attributed to many factors, such as low urease activity or slow growth. However, the most likely culprit is ineffective biofilm formation of the strain, since it has been shown that increased production of exopolysaccharide can greatly increase MICP rates (Hoffmann et al. 2021).

Despite the initial failure, we observed an interesting phenomenon when the two strains (DSM 33 and ANT_H38) were combined. If a 1:1 mixture of both microorganisms was used to inoculate the sand beds, a strong, synergistic effect was observed. The mixed approach resulted in strong biocementing effect, where the internal friction angle was comparable to that obtained by reference strain alone, but soil cohesion was almost four times greater. However, the exact mechanism underlying this synergy remains unknown. Given that MICP efficiency is influenced by various factors, such as pH, nitrogen availability, calcium concentration, and even soil particle size, pre-treatment of the soil with a metabolically distinct strain could create more favourable conditions for biocementation (Tang et al. 2020). Therefore, we hypothesise that the ANT_H38 strain may act as a pioneer, modifying the chemical environment and forming a biofilm that supports the growth of the other strain. This process could explain the observed synergy, even if only partially. Additionally, the first strain may facilitate calcium carbonate precipitation by providing nucleation sites, or metabolising inhibitory compounds that would otherwise hinder the process (Fu et al. 2023; Haystead et al. 2024). A change in the morphology of formed calcite crystals should also be considered, given that soil cohesion was the only heavily affected factor between reference strain and two-strain system measurements (Tang et al. 2020). Given the complexity of the process, it is difficult to pinpoint the exact cause of the observed synergy, and further research is needed to fully understand the underlying mechanisms.

Overall, our results point towards a plethora of possible implementations of such a two-strain system. Most prominent include soil stabilisation for construction industry, as well as prevention of soil erosion and desertification processes (Qabany and Soga 2013; Zhang et al. 2021). In combination with the unique ability of *Sporosarcina* sp. ANT_H38 to grow under low temperature conditions, many previously inaccessible opportunities, such as year-round application of MICP-based methods in cold and moderate climatic zones become open.

In this work, we showcase the enhancement of the biocementation process achieved via a combination of *Sporosarcina* sp. ANT_H38 — a urease-producing psychrotolerant, and *S. pasteurii* DSM 33 — the type strain for most biocementation-related experiments. Our results clearly indicate the strongly beneficial effect of such an approach, representing a promising new approach in construction-related biotechnology. By utilising a psychrophilic strain, we introduce the ability to apply biocementation at low temperature, giving hope for year-round applications in temperate climatic zones. Overall, we indicate that *Sporosarcina* sp. ANT_H38 strain can be effectively applied as an enhancer strain for other strains capable of MICP.

## Supplementary Information

Below is the link to the electronic supplementary material.

Supplementary file1 (PDF 250 KB)

## Data Availability

All data described in the manuscript are available either in Electronic Supplementary Materials associated with this manuscript or in genomic database. All of the data generated or analysed during this study are included in this published article, Electronic Supplementary Materials associated with this article or in the genomic database. The datasets supporting the conclusions of this article are available in the NCBI GenBank with ID GCA_046581305.1. *Sporosarcina* sp. ANT_H38 strain was deposited in the Polish Collection of Microorganisms (Wroclaw, Poland) with ID B/00547. All other experimental data is available from the corresponding author on request.
