# Supplementary material for: Genome-guided development of a bacterial two-strain system for low-temperature soil biocementation
Source: Appl Microbiol Biotechnol. 2025 Mar 18;109(1):66. doi: 10.1007/s00253-025-13448-8 (PMC11919988; doi:10.1007/s00253-025-13448-8)
Supplement: Supplementary file 1 — Supplementary file1 (PDF 250 KB) [file 253_2025_13448_MOESM1_ESM.pdf]

Genome-guided development of a bacterial two-strain system for low-temperature soil biocementation

Karol Ciuchcinski[1], Grzegorz Czerwonka[2], Przemyslaw Decewicz[1], Zofia Godlewska[1], Katarzyna Misiolek[3], Katarzyna Zegadlo[2], Michal Styczynski[1], Lukasz Dziewit[1,*]

[1] Department of Environmental Microbiology and Biotechnology, Institute of Microbiology, Faculty of Biology, University of Warsaw, Warsaw, Poland.

[2] Department of Microbiology, Institute of Biology, Faculty of Natural Sciences, Jan Kochanowski University, Kielce, Poland

[3] Department of Hydro–Engineering and Hydraulics, Faculty of Building Services, Hydro and Environmental Engineering, Warsaw University of Technology, Warsaw, Poland

* Corresponding author:

Lukasz Dziewit; phone: +48 225542004; e-mail: l.dziewit@uw.edu.pl

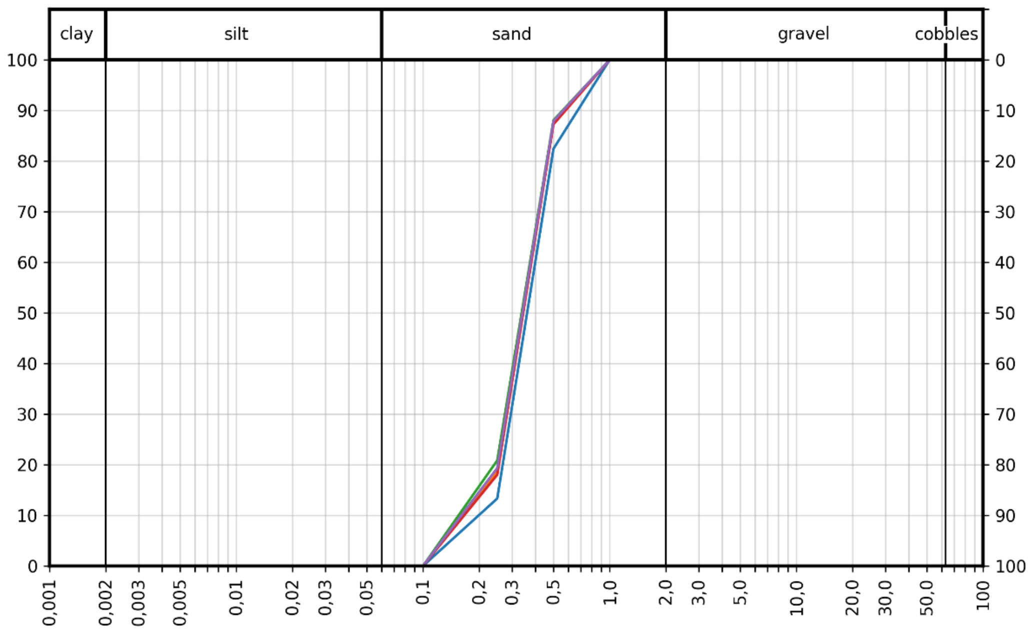

Figure S1. Sands grain-size distribution curves.

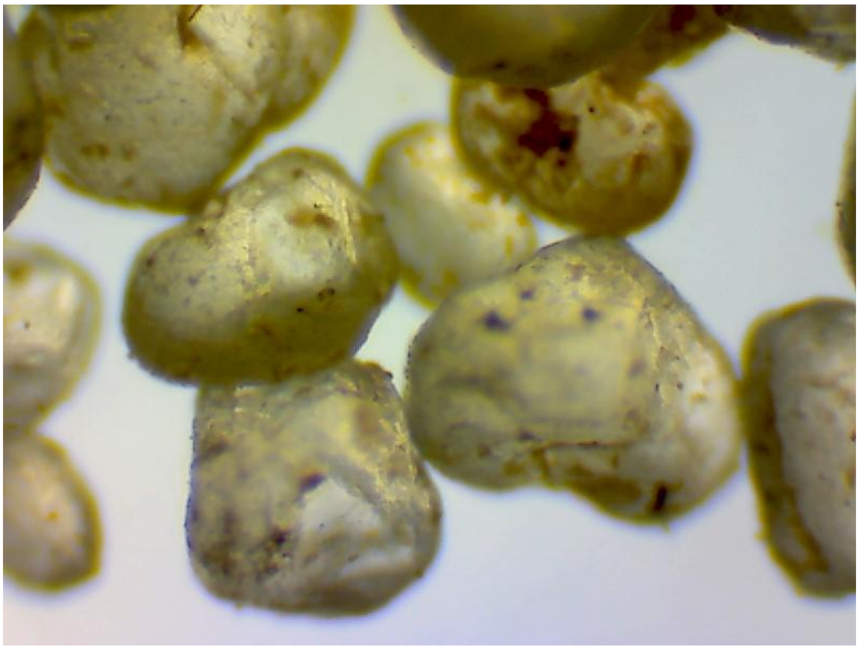

Figure S2. The grains of sand (marine origin) - light microscope photograph.

Table S1. General *Sporosarcina* sp. ANT_H38 genome characterisation.

**Feature**	**Calculation**
Number of contigs	5
Genome size (bp)	4,631,474 bp (chromosome);<br>6,243 bp (pA38H1); 10,106 bp (pA38H2); 10,532 bp (pA38H3); 27,152 bp (pA38H4)
GC content for chromosome (%)	39.9
Number of genes	4,512
Number of proteins with functional assignments	3,785
Number of proteins with EC number assignments	441
Number of tRNA genes	83
Number of regulatory RNA genes	104

Table S2. Genes involved in cold resistance identified within *Sporosarcina* sp. ANT_H38 genome.

**Locus tag**	**Gene/protein name**	**Function**	**Reference database**
GGGNBK_00030	*rplK*	50S ribosomal protein L11	CAPP database
GGGNBK_00170	unknown	hypothetical protein	CAPP database
GGGNBK_00175	*acoD*	Acetaldehyde dehydrogenase 2	CAPP database
GGGNBK_00790	*ureA*	Urease subunit gamma	CAPP database
GGGNBK_01250	*rpmH*	50S ribosomal protein L34	CAPP database
GGGNBK_01255	*dnaA*	DNA binding and replication initiator, global transcription regulator	Barria *et al.*
GGGNBK_02325	unknown	hypothetical protein	CAPP database
GGGNBK_04775	unknown	hypothetical protein	CAPP database
GGGNBK_05395	unknown	hypothetical protein	CAPP database
GGGNBK_05755	*icl*	Isocitrate lyase	CAPP database
GGGNBK_06320	*pseI*	Pseudaminic acid synthase	CAPP database
GGGNBK_07760	*sucD*	Succinate--CoA ligase [ADP-forming] subunit alpha	CAPP database
GGGNBK_08030	*nusA*	Transcription termination/antitermination/elongation L factor	Barria *et al.*
GGGNBK_08045	*infB*	Protein chain initiation factor IF2, translation initiation, fMet-tRNA binding, protein chaperone	Barria *et al.*
GGGNBK_08075	*pnp*	3′–5′ exoribonuclease; component of RNA degradosome; cold shock protein required for growth at low temperatures	Barria *et al.*
GGGNBK_08180	*recA*	General recombination and DNA repair; induction of the SOS response	Barria *et al.*, CAPP database
GGGNBK_10195	*cspB*	Cold shock-inducible; function unknown	Barria *et al.*, custom cspABC database
GGGNBK_10335	*sodA*	Superoxide dismutase [Mn]	CAPP database
GGGNBK_11075	unknown	hypothetical protein	CAPP database
GGGNBK_11195	*katA*	Catalase	CAPP database
GGGNBK_11645	unknown	hypothetical protein	CAPP database
GGGNBK_11825	unknown	hypothetical protein	CAPP database
GGGNBK_11850	*cspA*	Cold-inducible RNA chaperone and anti-terminator; transcriptional enhancer	custom cspABC database

GGGNBK_14565	unknown	hypothetical protein	CAPP database
GGGNBK_15425	unknown	hypothetical protein	CAPP database
GGGNBK_15580	*infC*	Protein chain initiation factor IF3, translation initiation, stimulates mRNA translation	Barria *et al*.
GGGNBK_17365	unknown	hypothetical protein	CAPP database
GGGNBK_17800	*menB*	1,4-dihydroxy-2-naphthoyl-CoA synthase	CAPP database
GGGNBK_18125	unknown	hypothetical protein	CAPP database
GGGNBK_19845	*clpP*	ATP-dependent Clp protease proteolytic subunit	CAPP database
GGGNBK_20925	*purE*	N5-carboxyaminoimidazole ribonucleotide mutase	CAPP database
GGGNBK_22000	unknown	hypothetical protein	CAPP database
GGGNBK_22350	*cspA*	Cold-inducible RNA chaperone and anti-terminator; transcriptional enhancer	custom cspABC database
GGGNBK_22900	*rpsK*	30S ribosomal protein S11	CAPP database
GGGNBK_22915	*infA*	Protein chain initiation factor IF1, translation initiation	Barria *et al.*, CAPP database
GGGNBK_22940	*rpsE*	30S ribosomal protein S5	CAPP database
GGGNBK_22960	*rpsZ*	30S ribosomal protein S14 type Z	CAPP database
GGGNBK_22975	*rplN*	50S ribosomal protein L14	CAPP database
GGGNBK_22990	*rplP*	50S ribosomal protein L16	CAPP database
GGGNBK_23005	*rpsS*	30S ribosomal protein S19	CAPP database
GGGNBK_23030	*rpsJ*	30S ribosomal protein S10	CAPP database
GGGNBK_23040	*tufA*	Elongation factor Tu	CAPP database
GGGNBK_23055	*rpsL*	30S ribosomal protein S12	CAPP database

Table S3. Selected genes and their predicted functions (excluding replication and mobilisation for conjugal transfer) found within the ANT_H38 plasmids.

**Locus tag / gene name**	**Plasmid**	**Predicted protein**	**Putative function**
GGGNBK_23135	pA38H2	Restriction endonuclease	Restriction/modification system
GGGNBK_23150	pA38H2	N-acetylmuramoyl-L-alanine amidase	Biofilm formation modulation/phage defense
GGGNBK_23155	pA38H2	MerR family transcriptional regulator	Metal-dependent metabolism regulation
GGGNBK_23210	pA38H3	Carbonate dehydratase	pH regulation
GGGNBK_23180	pA38H3	Ktr system potassium transporter B	Potassium uptake/osmotic stress resistance
GGGNBK_23195	pA38H3	MerR family transcriptional regulator	Metal-dependent metabolism regulation
GGGNBK_23260	pA38H4	MerR family transcriptional regulator	Metal-dependent metabolism regulation
GGGNBK_23275	pA38H4	Putative triple helix repeat-containing collagen	Spore formation
GGGNBK_23295	pA38H4	ABC transporter	ATP-binding protein
GGGNBK_23300	pA38H4	UBA/THIF-type NAD/FAD binding protein	Thiamine biosynthesis
GGGNBK_23350	pA38H4	MerR family transcriptional regulator	Metal-dependent metabolism regulation

Table S4. Insertion sequences identified within the *Sporosarcina* sp. ANT_H38 genome.

Locus tag(s)	Coordinates	IR	DR	IS family (according to ISfinder database)
GGGNBK_01305	273848 - 275021	IRL:AAATGCAACAAC<br>IRR:GTTGTTGCATTT	AAT	IS*30*
GGGNBK_02100,<br>GGGNBK_02105	438906 - 440642	IRL:ACGAGGACACTGAAAAA<br>IRR: TTTTTCAGTGTCCTCGT	-	IS*1182*
GGGNBK_02205	457357 - 457662	Unknown	Unknown	IS*150*
GGGNBK_04295	903872 - 905443	Unknown	Unknown	No significant similarity to other transposases from ISFinder DB
GGGNBK_05415	1114155 - 1114442<br>(complement)	Unknown	Unknown	No significant similarity to other transposases from ISFinder DB
GGGNBK_08435	1754374 - 1755276<br>(complement)	Not found	Not found	IS*L3*
GGGNBK_09275,<br>GGGNBK_09280	1947278 - 19438346	IRL:AAAATAAAAAAGTC,<br>IRR:GACTTTTTTATTTT	Not found	IS*150*
GGGNBK_09340,<br>GGNBK_09345	1958701 - 1960420<br>(complement)	IRL:GGACGCTTTCCGCGGGCACGGC<br>IRR:GCCGTGCCCGCGGAAAGCGTCC	GAT/GCT	IS*L3*
GGGNBK_12810	2618952 - 2619736<br>(complement)	Not found	Not found	IS*1182*
GGGNBK_14925	3094101 - 3094502<br>(complement)	Not found	Not found	IS*21*
GGGNBK_17765	3705396 - 3705704<br>(complement)	Unknown	Unknown	No significant similarity to other transposases from ISFinder DB

Table S5. Genes responsible for carbohydrate metabolism identified uniquely in *Sporosarcina* sp. ANT_H38 genome.

**Gene name**	**Function**	**Loci name**
araA	L-arabinose isomerase	GGGNBK_13170
araB	Ribulokinase	GGGNBK_13175
araC	Arabinose operon regulatory protein	GGGNBK_00325, GGGNBK_08640
araP	L-arabinose transport system permease protein	GGGNBK_13150
araQ	L-arabinose transport system permease protein	GGGNBK_13125, GGGNBK_19645
bga	Beta-galactosidase	GGGNBK_13295
bglK	Beta-glucoside kinase	GGGNBK_16080
bglY	Beta-galactosidase	GGGNBK_08660
crr	PTS system glucose-specific EIIA component	GGGNBK_00395, GGGNBK_22850
iolE	Inosose dehydratase	GGGNBK_13195, GGGNBK_13410
iolG	Inositol 2-dehydrogenase/D-chiro-inositol 3-dehydrogenase	GGGNBK_13270
larA	Lactate racemase	GGGNBK_02645
licH	putative 6-phospho-beta-glucosidase	GGGNBK_16075
malG	Maltose/maltodextrin transport system permease protein	GGGNBK_08655
malP	PTS system maltose-specific EIICB component	GGGNBK_00385
malX	PTS system maltose-specific EIICB component	GGGNBK_08590
melA	Alpha-galactosidase	GGGNBK_13070,GGGNBK_13075,GGGNBK_13080
melD	Melibiose/raffinose/stachyose import permease protein	GGGNBK_13885, GGGNBK_22850
mngR	Mannosyl-D-glycerate transport/metabolism system repressor	GGGNBK_16130
yteP	putative multiple-sugar transport system permease	GGGNBK_13475
